# ‘Buying Salad Is a Lot More Expensive than Going to McDonalds’: Young Adults’ Views about What Influences Their Food Choices

**DOI:** 10.3390/nu10080996

**Published:** 2018-07-30

**Authors:** Eloise Howse, Catherine Hankey, Margaret Allman-Farinelli, Adrian Bauman, Becky Freeman

**Affiliations:** 1 Prevention Research Collaboration, Sydney School of Public Health, Faculty of Medicine and Health, University of Sydney, Level 6 Charles Perkins Centre D17, Johns Hopkins Drive, Sydney, NSW 2006, Australia; Adrian.bauman@sydney.edu.au (A.B.); becky.freeman@sydney.edu.au (B.F.); 2 School of Medicine, Dentistry and Nursing, University of Glasgow, New Lister Building, Glasgow Royal Infirmary, Glasgow G4 0SF, UK; Catherine.Hankey@glasgow.ac.uk; 3 School of Life and Environmental Sciences, Faculty of Science, University of Sydney, Level 4 Charles Perkins Centre D17, Johns Hopkins Drive, Sydney, NSW 2006, Australia; Margaret.allman-farinelli@sydney.edu.au

**Keywords:** young adults, food choices, food environments, attitudes, prevention, Australia, Scotland

## Abstract

Young adults (18–30 years of age) are an ‘at-risk’ group for poor dietary behaviours and less healthy food choices. Previous research with young adults has looked at the barriers and enablers driving their food choices, focusing primarily on university and college students. However, there is less research using qualitative methods with young adults as a broader population group. This study aimed to explore the experiences of young adults in two different yet similar settings: Sydney, Australia and Glasgow, Scotland. Eight focus groups of young adult participants, ranging in size from 2–6 participants, were held in Sydney, Australia (*n* = 14) and Glasgow, Scotland (*n* = 16) to discuss, explore and compare the determinants and influences of their food choices. Focus group transcripts were coded thematically based on a process of narrative analysis. Three major narratives were identified across both locations: value of food; appeal of food; and emotional connections with food. These narratives were underpinned by a broader narrative of ‘performing adulthood.’ This narrative reflected a belief amongst participants that they should make rational, informed choices about food despite this conflicting with their broader food environment. Future research could examine which environment-level or policy-based interventions are most acceptable to young adults in terms of influencing their food choices and dietary behaviours.

## 1. Introduction

Improving diet is critical to preventing chronic and non-communicable diseases (NCDs). The dietary recommendations of the World Health Organization [1] encourage: consumption of a variety of fruits, vegetables, whole grains, legumes and nuts; and limiting consumption of ‘discretionary’ food and beverage items—products that are high in fats, salt and/or added sugars (including sugar-sweetened beverages). Globally, poor dietary factors (when combined) account for 9.6% of attributable disability-adjusted life years for both men and women and are responsible for 18.8% of deaths worldwide [2]. Poor diet is also strongly interrelated with high body mass index (BMI); this has associated consequences for other major NCD risk factors such as high blood pressure [2]. 

Poor dietary behaviours are of particular concern for young adults, as the transition from adolescence to adulthood is a critical period for influencing both short and longer term NCD-related health risks and outcomes [3,4]. For example, current research suggests young adults are experiencing rapid weight gain in part due to changing dietary behaviours [5,6,7,8,9]. However, the causes of poor diet are complex. Changes in local, national and global food systems towards energy-dense, industrially-processed foods and drinks have been identified as major components of ‘obesogenic’ environments [10,11,12]. People’s decisions and choices about food are influenced by diverse factors operating at multiple levels including at the individual, social, community and environment level [13,14]. People’s agency in determining their food choices shifts depending on these changing macro-level and micro-level contexts, which also change during different life stages and trajectories [15]. 

The scientific literature on young adults’ food choices has focused mostly on the barriers and enablers to healthy eating for young adults. The barriers experienced by young adults in terms of making different food choices can be driven by specific life stage changes, such as moving from school and family-based environments to starting university and college [16,17]. First year university in particular has been associated with poor eating practices and weight gain [9]. Other research has identified the following themes, such as: time is a major barrier for young adults in making healthier food decisions [18]; young adults prioritise foods which taste good, are convenient and low in cost [19]; and young adults are aggressively marketed to through advertising and promotions of discretionary foods particularly on social media [20]. 

A systematic review of food choices among young adults [8] categorised the barriers and enablers they experienced into three themes: food security and convenience (physical and environmental factors); intrapersonal (individual factors); and interpersonal (social or situational factors). This review identified twelve barriers to healthy eating, including cost of food, perceived lack of time for preparing healthier meals and specific emotional responses towards less healthy foods. However, 70% (24/34) of the reviewed studies focused on university or college student populations and the review only included studies with young adults aged 18–24 years. In addition, less than half of the reviewed studies used qualitative methods such as focus groups or semi-structured interviews and none of the studies utilised a comparative approach between different countries.

Another review found there were 105 factors that influenced the changing eating behaviours of young adults in the transition from adolescence to adulthood [21]. This review classified the factors into different levels of the socioecological model. It indicates that most research has focused on university or college students and individual-level factors relating to their food choices; in comparison, there is very little research on young adults as a broader population group and the influence of macro-environment and policy-level factors. 

Given these gaps in the literature, this study used a constructionist approach to explore young adults’ views about the determinants of their food choices. A constructionist approach to food and diet suggests that the agency of individuals in making decisions about food operates in conjunction with broader macro-level structures, meaning that different people will interpret and define their experiences in different ways [15]. This approach is also echoed by the socioecological model of health [22,23] and social cognitive theory [24], which both identify a reciprocal relationship between multiple factors at the individual, societal and environmental level that influence health behaviours, including food and dietary behaviours. Exploring how young adults frame and discuss their food choices and preferences in the context of their physical and social environments and settings is crucial to understanding what factors are driving their behaviours and decision-making. 

This study aimed to explore the experiences of young adults as a broader population group in two different yet similar settings: Sydney, Australia and Glasgow, Scotland. Though differing in some aspects, such as population size, climate and levels of social deprivation, both cities are similar in terms of their urbanised food environments with fast food outlets, supermarkets and convenience stores which mean easy access to discretionary food products. These countries also have similar challenges relating to dietary behaviours within their young adult populations. In Australia, young adults have low consumption of fruit and vegetables [25] and are reported to consume larger amounts of added sugar compared to older adults [26]. Young adults’ intake of discretionary foods as a proportion of total energy intake is also higher than other age groups [27]. In Scotland, the picture is similar: 20% of the energy in Scottish diets comes from discretionary foods with younger adults consuming fewer fruit and vegetables than older adults [28,29].

This study aimed explore the experiences of young adults in two geographic settings to find out whether these experiences of food choice are shared, indicating possible applicability or transferability with other urbanised young adult populations in high income countries. 

## 2. Methods 

Young adult participants between 18 and 30 years of age living in Sydney, Australia and Glasgow, Scotland, were recruited through targeted social media posts and online advertisements to participate in a 1 hour focus group. Participants were asked to complete a short screening questionnaire and provide their first name, gender, current employment or study status (whether they were full time employed, part time employed, full time student, or other), as well as highest level of education attained. Eligibility criteria was age (18–30 years) and currently living in Sydney or Glasgow. Consent to participate was gained through this screening survey. 

Participants were purposively allocated to focus groups based on age, gender and availability to ensure a breadth of ideas and experiences. Each focus group aimed to be evenly split with equal numbers of men and women, as gender was not a main focus of this study. However, the focus groups aimed to have different combinations of ages, for example having separate groups of younger (18–24 years of age) and older adults (25–30 years of age) as well as a combined group of young adults ranging from 18 to 30 years of age. Focus groups were held in centrally-located, inner city venues in Sydney and Glasgow in August–October 2017 and facilitated by the primary author, a trained focus group facilitator and a young adult researcher. Verbal and written consent was confirmed at the beginning of each focus group. All focus groups were audio recorded for the purposes of analysis. Each focus group participant received a cinema voucher.

Focus group questions were developed from the research question and followed the structure suggested by Liamputtong [30] which involves beginning with introductory questions, followed by transition questions leading to the focus questions based on the research questions. Each focus group involved a semi-structured exploration of the following topics: identifying participants’ favourite food or drink; what influences the participants to purchase that food or drink; canvassing their views about policy options to address diet; perceived differences between children and adults as it related to those policy options and making food choices; and what could be done to assist young people in making different choices about food. This paper focuses primarily on the first two topics. 

A narrative constructionist approach was used to analyse the data. This approach examines personal narrative as part of a wider social process [31] and was chosen in order to reflect the complexities of food choice [14]. Narrative analysis can also apply to smaller narrative fragments that help people to make sense of complex factors at play in influencing people’s health [32]. Narrative-based research can also shed light on how individuals make sense of and elucidate meaning from events [33].

The process of narrative analysis included transcript review, coding of narratives, story or narrative creation and analysis of narratives [34]. Transcripts and focus group notes were coded into specific thematic stories, which were then grouped together to form three main narratives, supported by a broader narrative. This analytical process was iterative and ongoing during the process of data collection to ensure ‘conceptual density’ [35] was reached, in which sufficient depth was gained from the data to build a relevant theoretical framework. Focus group transcripts were thematically analysed and coded iteratively by one researcher then cross-checked with a secondary researcher, with regular discussions and note-sharing between the researchers after each focus group. These narrative codes were then developed into the four broader narratives which were reviewed by all five authors. Pseudonyms were given to study participants in line with research ethics. This research was approved by the University of Sydney’s Human Research Ethics Committee (project number 2017/579) and the University of Glasgow’s College of Medical, Veterinary and Life Sciences Human Ethics Committee (project number 200170012).

## 3. Results and Discussion

One hundred and ninety-eight young adults completed the screening survey. From this, a total of thirty young adults aged between 18 and 30 years of age were allocated to eight face-to-face focus groups, three in Sydney (*n* = 14) and five in Glasgow (*n* = 16). The characteristics of the focus group participants are summarised in Table 1. The size of each focus group was kept small, 4–6 participants per group, as has been recommended by some qualitative researchers in health [30,36]. However due to last minute drop-out of participants in Glasgow beyond the control of the researchers, some of the Glasgow groups had fewer participants than other groups.

**Figure 1:**
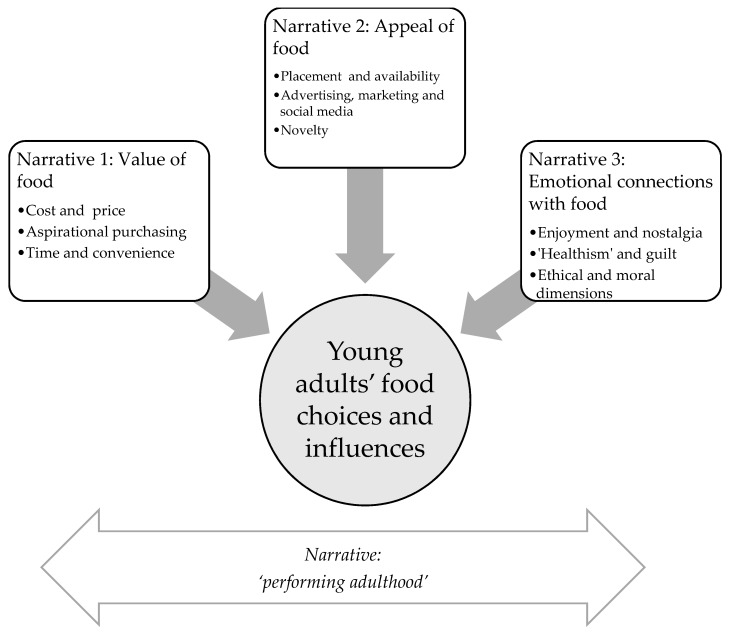
Narrative and thematic framework.

Three main narratives, shared across both locations, were developed from the focus group data: value of food; appeal of food; emotional connections with food. These narratives were developed from a number of narrative codes or thematic stories relating to specific influences of food choice which participants referred to in their discussions. While many more narrative codes were developed and analysed than have been included in the results and discussion (see Supplementary File, Table S1), we prioritised those narrative codes that were in line with the theoretical framework and approach and which best represented the views of young adults about their food choices. The three main narratives were supported by an overarching narrative about the perceived need to behave as a ‘good’ adult and adequately ‘perform adulthood’ in relation to food, shown in Figure 1. 

### 3.1. Value of Food

#### 3.1.1. Cost and Price

Participants in both Sydney and Glasgow reflected a common finding relating to food and young adults: that price significantly influences their food choices [8,19,39]. However, our participants also reflected a range of ‘value negotiations’ relating to food. Value negotiations involve considering and weighing up the value of decisions at that point in time [15]. For our participants, this involved negotiating the perceived value of certain foods in terms of price, the relationship with time and convenience and in terms of flavour and taste. The perceived higher cost of ‘healthier’ foods is often seen by young people as a major barrier to making different decisions around food [39]. Michael (26 years, male) identifies the perceived need to ‘eat healthy’ but the challenges this brings in terms of affordability for a young person:

*Michael: (...) I think it’s really really expensive to eat healthy. I’ve tried to be on a diet since Easter, or to even sometimes eat organic. It’s—it’s impossible. Um, you know, even if you work and you’re on a scholarship, it’s impossible, let alone someone that might not have an income, or you know, on Centrelink (income support) or have a very basic job. It would be impossible to maintain that, especially with the rising housing prices*.

Michael’s narrative identifies two important factors at play in determining food choice: first, the lower price of less healthy foods as opposed to (perceived) healthier, fresh foods they feel they should be eating; and second, the challenges currently facing young adults in their particular life stage, such as studying full time and working in low wage jobs (including internships). Being on a low income while studying or undertaking further training is a shared and agreed narrative amongst our young adult participants, which affects their agency to choose certain foods, particularly healthier foods. 

#### 3.1.2. Aspirational Purchasing

While half of our participants identified as full-time university students, other participants perceived and negotiated the cost and value of food differently, depending on whether they had entered a key stage within young adulthood: entering the full-time workforce. According to some participants, this transition allows them to purchase more expensive and enjoyable foods that are associated with a higher value. Amanda (25 years, female) and George (28 years, male) exemplifies this view—they place their food choices within a chronological narrative of moving from university study to full time employment:

Amanda: Um, ah, the only thing I would think is that price, I’ve noticed, since stopping university and starting working full time, that disposable income has now meant that I don’t really consider price in what I want to eat anymore. It’s way more just—and that sounds terrible—but it’s just what I feel like. And I totally acknowledge I’m fortunate to do that but yeah I think it’s quite dependent, because I know that when I was a student it was much more driven by whether or not I thought that was a reasonable price to pay for whatever it was I wanted, whereas now I’m very indulgent … yeah (laughs).

George: Yeah I’ve found that as well. I’ve sort of transitioned between sort of, staff and student depending on which year I’m in and so the disposable income really, sort of, particularly the last year when I was in full time work, it meant that yeah I could just indulge cos there was just that much more income.

This narrative suggests that some young adults aspire with their new found disposable income to ‘indulge’ in more expensive or enjoyable foods, though it was unclear whether ‘indulgence’ referred to healthier or less healthy foods. So, while income may be a barrier to purchasing certain foods during university or college study, young adults also aspire to purchase and choose foods that reflect the next stage in adulthood of full time work and disposable income. Neely et al. discuss how young people negotiate their independence through their food practices, particularly with friends and new social groups [40]. In our study, the participants shared a narrative of aspiration in which the ability to make different food choices reflected their negotiation of autonomy and independence as individuals and adults.

#### 3.1.3. Time and Convenience

The financial cost of food is not the only factor relating to young adults’ value negotiations of food. Our participants’ views were shaped by perceptions about time and convenience as they relate to food and health, as previously outlined by other research [41,42]. Some of our participants identified that ‘easy’ and ‘quick’ foods, which cost less in terms of time, are often prioritised by people on lower incomes. In our participants’ eyes, the expense of time is as crucial as the economic expense, as Amber (20 years, female) explains:

Amber: (…) cos healthy foods, unless you’ve got the time to prepare them—a fully prepared healthy meal is quite expensive. It’s not expensive if you’re buying your fruit and veg and cooking at home but especially like people on lower incomes, they don’t have the time every night to prepare a fully healthy meal. And if they need to buy cheap stuff that they can get quickly to keep their families alive, I think that’s more important than being healthy for that time being.

Amber’s narrative reflects a conscious prioritising of foods that are filling and have a time value rather than a health value. Young adults are aware that more convenient foods tend to be less healthy and may actually be costlier in the short and longer term. Time is therefore an essential factor to consider when negotiating the value of their food choices. 

### 3.2. Appeal of Food

#### 3.2.1. Placement and Availability

Participants in Sydney and Glasgow were aware of the range of techniques used to make specific food choices more appealing, such as the placement of snack food displays in supermarkets [43] and the availability and accessibility of food outlets in specific areas [44]. Some participants saw supermarkets as trying to manipulate people to buy less healthy food products that they didn’t necessarily need or want. This was characterised by participants like Rebecca (25 years, female) as ‘impulse buying’:

Rebecca: I’m susceptible to impulse buying and it’s always the things that are bad for you that are around at the tills and last minute you grab them. There’s no like apples stacked next to the cash register and things. But there’s lots of layouts at supermarkets and things, the way they plan it for people to walk through, it’s all structured so you buy what they want you to buy kind of thing. Like they put the bakery section at the back, I’ve heard the smell of the bread is meant to lure you through. I don’t know how true that is but if you look at any supermarket they usually are right at the back and you can smell it.

This was a shared experience with other participants, who were aware of how the physical environment shapes their food choices, particularly in specific food environments like supermarkets and convenience stores. Placement and availability of certain foods was particularly effective when combined with price, as Hunter (22 years, male) and Mia (22 years, female) identify in terms of supermarkets:

Hunter: Yeah, they’re very tricky. They know exactly how to get you. Put all the chocolate on the counter and then you’re like, ‘Oh, well, I might as well just grab a thing.’

Mia: Yeah, because they put the little bars—it’s only a dollar, you’re like, ‘Oh, I’m spending this much on groceries. What’s a dollar?’

This approach of ‘why not?’ when making food choices was echoed by other participants in Sydney and Glasgow. It suggests that young adults feel unable to reject the impulsive buying practices encouraged by the broader food environment, regardless of how educated or willing they may be to do so. 

#### 3.2.2. Advertising, Marketing and Social Media

Our participants also mentioned the role of advertising and marketing to increase the appeal of certain foods. They noticed that particular foods and brands were heavily marketed through social media, a common advertising technique particularly for younger audiences [45]. For example, Lily (19 years, female) noted that:

Lily: (…) people are impressionable, especially kids. Like, if you see something all the time then you’re more likely to want it, rather than if it’s something you don’t know. Like, you’re more likely to go buy Coca-Cola rather than like Lidl-owned coke or something as a kid, then you’ll be like ‘I want the normal Coco Pops,’ not the coca pops or whatever they are. It’s just, you’re going to see it a lot and so I don’t feel like it’s a great idea when like celebrities are sponsoring it, cos then like on Instagram and stuff you see someone promoting something but they’re being paid to do it. And like I know they’ve now got that thing where you’ve got to say it’s an ad, before they had that and they thought like people could just be like, ‘yeah, I really love this’ but they’re being paid to do that. They might not actually like it but then kids are going, ‘oh, Kim Kardashian likes this, I’m going to have this.’

Lily suggests that the saturation of particular brands across social media and other avenues is a strong factor in driving young people’s choices around food. This is further compounded by the influence of a celebrity figure who promotes particular social norms and an idealised image associated with consumption. Other young adults were also aware of how social media sponsored and targeted advertising to them, as Sarah (25 years, female) complains:

Sarah: (...) I don’t really watch sports or things that are sponsored and I’m on social media all the time and it’s really annoying. Um, so yeah.

Moderator: What’s really annoying?

Sarah: The like—just ads for McDonalds or whatever. Um…

Moderator: So, they sort of, pop up, randomly or…

Sarah: Yeah. Or, like on Instagram you sort through and there is always just like a sponsored ad from wherever. Um, I had another thought but it’s completely gone now. Um, yeah, I have a problem I suppose with the whole idea of advertising swaying people to make unhealthy choices that the company themselves knows are unhealthy, but—that’s what they make and it employs a lot of people and they make money. Um but yeah, I think advertising especially is very detrimental to people’s education and ability to make good choices for themselves.

Sarah suggests that exposure to advertising and marketing provokes a major challenge for adults to make ‘good choices,’ regardless of whether they are aware of the message or not. For young adults, critical awareness of marketing of particular messages may not be a sufficient defence against the impact of the advertising [20]. This is particularly the case when celebrities are promoting specific products. However, there were differing accounts from our participants as to the impact of advertising. Some participants said they noticed advertising of specific food products but it didn’t affect their choices, while others said they felt influenced by it. Bandura suggests that individuals perceive and experience the interaction with their environment differently, which produces different experiences of self-efficacy in terms of health behaviours and decisions [24]. This may explain why some young adults in this study felt like they had more control over their food choices compared to their peers, particularly in regard to the effect of advertising. 

#### 3.2.3. Novelty

In conjunction with placement and advertising, the novelty of new products played an important role in making certain foods more appealing than others through cultivating interest and engagement. Some young adults like Charlie (25 years, male) referred to the excitement of seeing or trying new products:

Charlie: It’s the excitement when you get—if you see something that you really like and it’s your favourite, you will get excited, you will get passionate. And then you will get like over excited at times. So I think if it does look good, you will pick it up and you get excited. But I think if it’s your favourite food and you look and it doesn’t look as good, you wouldn’t pick it up. And you know sometimes—I know I start thinking, ‘oh, I might change my favourite,’ or I might change my options next time. That’s just the way my brain works, it gets creative. So if that doesn’t look good, I’ll try something else. Um, I think as well, if you buy sort of a same product—if you just buy the same potato salad every time—you will get bored of it and you will get excited if, say, a new—say, a chilli potato salad—comes out, or something different comes out. You will continue to buy your sorta regular one but you will be excited to pick up a new one.

Our participants were heavily influenced by the perceived need to ‘try’ something new. Sometimes, the novelty aspect of a product was more influential than price on their food choices. Young adults on lower incomes could be influenced by brands releasing and promoting newer products, as Lily (19 years, female) outlines:

Lily: Mostly that it’s cheap at the moment, because I’m studying and I’m still looking for a job. So it’s not, um… like, there’s certain stuff that I will pay more for, or if I know it’s expensive then I’ll just avoid it. But like sometimes you see that something’s coming out and then you want to go by that. Like, Ben & Jerry’s have released vegan, dairy-free ice cream now in the UK. So that costs like, £5.50 for a tub, which was kind of like, ‘oh, do I really want to pay it for that?’ Because you can get one of the normal ones for like £2.50. But then I wanted to try it.

Moderator: So how did you find out about it? How did you find out about the Ben & Jerry’s thing, for example?

Lily: Um, Facebook—social media. Cos there was a lot of buzz about it cos then vegans can have it. So like, a lot of buzz around food seems to come when it’s good for the environment or it’s vegan friendly or it’s gluten-free or something. Something that like a specific group of people can have, then it gets a lot more buzz than if it’s something that everyone can have…I dunno.

Participants understood the techniques used by food companies and brands to promote their new products. These included the use of social media for ‘guerrilla’-style marketing campaigns [46] and the adoption of social movements (such as animal rights and environmental sustainability) to sell the new food product.

### 3.3. Emotional Connections with Food

#### 3.3.1. Enjoyment and Nostalgia

Many of our participants viewed food as a source of emotional connection and enjoyment. For some, the food they enjoyed most was based on recreating a connection to an idealised memory of a certain time or place, as Brayden (23 years, male) suggests:

Brayden: So I suppose I did a lot of travelling in that part of the world so I think that part of it is that it reminds you of travelling. It’s like nostalgic almost—not nostalgic in that same way but it brings you back there and it makes you feel you’re kind of connected to that travelling experience but that is definitely part of it. I really like also the idea of street food, so that part of Asia there’s loads of great street food. And, again, you can get that sort of approximation of that something in Glasgow. And other places you can approximate that street food feel even if it’s in a restaurant. So that, again, draws me to that kind of small portions but like me trying a few different things that really appeals to me and getting those sort of different flavours, different tastes and things like that is nice.

Other young adults talked about nostalgia in terms of the connection to childhood memories and reliving past experiences that were enjoyable, which Summer (26 years, female) identifies:

Summer: I’m a mixture between I like things that are familiar in a lot of ways. So I love—there’s some comfort foods like a Cadbury’s mini roll would be, when I was a kid that would be like a go-to (laughter)—you find something that’s familiar and has memories attached. But then there’s a kind of another part of the spectrum where I kinda—I just like finding new things. If there’s something new in the biscuit aisle I’ll probably have a try of it, at least once. So I think I’m less affected by the packaging, I’m more by past experience—trying new experiences I guess.

For young adults like Brayden and Summer, enjoyment of food was based not just on how it tasted but the circumstances in which the food was enjoyed and what those circumstances personally represented to them. Experiencing and enjoying new foods was thus influenced by memories of the past, though there seemed to be a balancing act between the familiar and the new.

#### 3.3.2. ‘Healthism’ and Guilt

Some participants talked about less positive emotional connections with food, such as the notion of ‘feeling bad,’ particularly in relation to the ‘healthy eating’ ideal, as Leah (19 years, female) identifies, albeit in a light-hearted way:

Leah: Yeah, I think I just like it because it tastes good. (laughter) Bad reason, yeah and also like convenience, especially since coming to uni, it’s become like a much bigger thing. Whatever takes the least time, yeah.

Moderator: What is it—why is it bad that it tastes good?

Leah: I don’t know, cos you should be eating food that’s you know, healthy, thinking about your nutrition and I’m just like, ‘I feel like chocolate,’ so you know (laughs) I don’t think about it too deeply.

In this narrative, there is a perceived difference between short term pleasure (of enjoying foods you ‘feel’ like) and long-term gain (of making the ‘right’ choice for your health). This was expressed by other (female) participants such as Natalie (23 years old, female) who link food with the notion of an ‘ideal weight’:

Natalie: It’s hard because I’m like, I have this mindset which is like what if I, you know, just kinda like, I want to enjoy myself and this would give me pleasure so like I should just eat it. But then I need to be like, ‘no,’ because that’s going to give me short-term pleasure. But in the long term, like, you know, getting to an ideal weight and being healthier is better for me, so I just need to persevere with that mindset. But I’m very much…also I think I’ve been told as well that I can eat a lot of sweet things. Whereas my boyfriend he’ll maybe have a few, like a couple of biscuits, whereas like I could finish the whole packet, no problem and I wouldn’t feel sick, whereas that would make him feel ill. So like I need to…moderation is what I need.

Young women like Leah and Natalie suggest that as adults they are now expected to make the ‘right’ and rational decision when it comes to food, which involves prioritising healthier foods and moderating their food choices, even though the cost to them is reduced pleasure and enjoyment or even negative feelings such as guilt and shame about the foods they usually enjoy. It reflects findings of other research that suggests young adults who engage in particular self-regulatory behaviours may enable healthier food choices [8,19]. However these types of attitudes might also reinforce normative and gendered expectations or ideals relating to food. This could inadvertently promote stigma around weight gain and food and encourage the view that individuals are responsible for behaviour change [47,48].

#### 3.3.3. Ethical and Moral Dimensions

Young adults in our focus groups demonstrated the importance of thinking about the broader systematic drivers and consequences of their food choices, such as sustainability and vegetarianism. They were highly conscious of the effects of their food choices and knowledgeable about the complexity of modern food systems as it relates to the welfare of animals or the issue of climate change and environmental sustainability. However this knowledge was related to powerful emotions such as guilt already discussed earlier, as Ryan (29 years, male) identifies:

Ryan: Um, what really influences me in my opinion is like those Netflix food documentaries (laughter) and I’m like ‘Ohhh.’ Like seriously. (laughter) they make you feel so bad about everything. It’s like—it really changes the way I want to consume things. And yeah, as tacky as it sounds, it’s like—it actually works: ‘Ah, I think I should go vegan now’ (laughter).

Being able to choose foods and products based on an ethical or moral understanding was one way in which our young adult participants enacted agency. Ella (25 years, female) similarly identifies this as an important consideration, telling a story about Nestlé:

Ella: (…) Um, I try to boycott Nestlé still and that’s really hard.

Moderator: So how come Nestlé?

Ella: Nestlé said that water isn’t a basic human right and you should have to pay for it. They also had—the main reason is way back, when I say way back the ‘80s, ‘90s, they had this big baby formula—they started marketing formula milk in Africa to mothers and saying this is an affluent product, you can have formula milk and be better than breastfeeding mums. However they were selling it in places where the water wasn’t sanitary. So the babies were actually getting very sick because they were drinking dirty formula milk. Um and Nestlé were like, ‘not our problem’ and they took no responsibility for it. So there was this huge boycott. So yeah, I have an app called ‘Buycott’ which I use if there’s something which I don’t know if it’s Nestlé I can check it on that. You can also—I try and avoid palm oil. Being a vegan’s restrictive enough and then when you starting going ‘I’m not going to have palm oil’ you’re like ‘okayyyy’.

The story that Ella tells is not simply about the importance of consumer boycotts. Rather she is showing through her story that people’s individual decisions about food are limited by the very nature of the food system itself, which she sees as being predominantly driven by large, powerful, multi-national food corporations. Trying to maintain one’s agency within such an environment is challenging. Ella suggests that apps and information may be limited in terms of affecting people’s food choices—that perhaps without addressing major structural drivers, it will continue to be difficult for other young consumers like her. The strongly held views about ethical and sustainable foods expressed in this study could be an important consideration for dietary interventions and policies targeting young adults, given the evidence suggesting positive attitudes in this area were associated with higher dietary quality amongst young adults [49]. 

### 3.4. Performing Adulthood

Underpinning these three narratives was a broader narrative about the challenges young adults experience in learning to ‘perform adulthood.’ Our participants identified the transition in early adulthood to new environments and settings that might reinforce or hinder their food choices. For example, the transition to university offers a number of competing demands for young adults which restricts their prioritisation of different food choices [17]. But our participants were also conscious of a conflict in the way they ‘ought’ to eat as adults (for example, prioritising healthier, less processed and more environmentally sustainable foods) versus what they ‘felt’ like based on the influences of their surrounding physical and social environments. This perceived need to make ‘good’ or healthier food choices was reinforced by the representation of what ‘adulthood’ meant to them: individual choice and rational decision-making. For example, some participants contrasted children’s and adults’ food choices, suggesting that children are more emotionally-driven compared to adults who are able (or should be able) to make more ‘informed choices’ whether or not to consume particular types of foods, as outlined by Sarah (25 years, female):

Sarah: I think it’s impulse and adults have—usually—a greater capacity to make rational, informed decisions. Yeah (…) I remember my mum wouldn’t want me to eat, I dunno, this chocolate bar. I’d be like ‘why not?’ And, the explanation was that it’s bad for you and as a kid you don’t know what that means. They just like ‘I don’t care! I don’t care if it’s bad for me!’ Whereas adults know that if you, like, eat that then there’s consequences and blah, blah, blah. Yeah, the kids don’t understand.

This message—of adults’ perceived ability to make to make their own decisions and ‘have more control’—was also represented in the way university was characterised as being different to school in regard to food choices, as Leah (19 years, female) identifies:

Leah: (...) I found at university people are more like—I don’t know, like, everyone’s pretty accepting, you know, you eat whatever you want to eat, or, um, some people—I guess because we have more control now than we did as kids—you’re able to decide your own diet and maybe you get into a certain diet, so I feel like we do have a bit more control. That’s quite exciting

Young people use food practices and choices to negotiate their independence and agency [40]. Our research suggests that the transition to adulthood provides opportunities for young adults to explore new food choices in the context of their new-found social, cultural and legal independence as adults. But our participants, regardless of city, also believed adults’ food choices were mostly influenced by personal control, willpower and knowledge. These narratives reflect findings from other research suggesting that some adult populations can believe strongly in notions of freedom of choice and individual responsibility while also acknowledging the influence of other factors [50,51]. However while some participants reflected these attitudes, other participants also noted that the environment had a powerful role to play in determining their food choices, as Ryan (29 years, male) indicates:

Ryan: at the moment I think, buying salad as a takeaway is a lot more expensive than going to McDonald’s. And if you can—I know, it’s probably…I don’t know how to solve it—but obviously fresh food is more expensive but if somehow that could be cheaper, maybe that’s more a better incentive to help people pick healthier food than the packaged confectionary.

Ryan’s narrative of comparing salad (perceived as a healthy food) and McDonalds (seen as a less healthy food) indicates he is conscious of how price influences people’s food choices and suggests these structural determinants should be addressed in order to help people make healthier food choices. These young adults, despite engaging with a narrative of adulthood and personal choices, still noticed that environments are challenging for people to overcome, even if they have both the will and knowledge to eat healthily, as Rebecca (25 years, female) says:

Rebecca: (…) It’s also, when you talk about convenience, um… I mean you’d go into a shop and if you’re in a rush, you’ve got 20 minutes for a lunch break, grab a wrap, a sandwich or something, a packet of crisps and a drink and you’d go. And if you look at that it’s full of sugar and salt. But you don’t have time to make something healthy. You can get a salad and things but a lot of these things, the salads, are packed with those sugars and salts anyway, so you don’t really have time to go and make your own fresh salads. I try and make my lunches in the morning but I can’t always and if I do I know where it’s come from, it’s just vegetables or whatever. But for convenience, I would always go for something less healthy.

Participants across both locations were highly aware of and well educated about what they ‘should’ be doing in regard to making food choices. However, these young adults struggled to put it into practice due to a competing number of narratives and sub-narratives, which they identified as driven by multiple influences at the micro- and macro-level of the broader food environment and system. These narratives and sub-narratives were commonly discussed by participants in both cities, suggesting there are some common challenges our participants experienced in adapting to adulthood, particularly as it relates to navigating the complexity of food choices. 

These results suggest there is a need to prioritise and implement policy-based and regulatory strategies for young adults that target both micro- and macro-level food environments and systems [10,52]. Other important strategies might involve taking a settings or systems-based approach to address food environments most affecting young adults, such as university food environments [19,53]. Strategies that address the wider environment in which young people make choices might enable and promote their sense of self-efficacy, thus allowing them to make healthier food choices [39]. Upstream, environment-level strategies may also provide ‘protection’ against unhealthy nutrition behaviours [12].

## 4. Limitations

There are a number of limitations to this research. The socioeconomic status of our participants suggests they were reasonably advantaged, living in a major urban centre and half were university or college students. Our participants were also not screened for dietary behaviours or health and nutrition literacy levels. Issues such as weight gain and body image were not canvassed or probed in the focus group discussions. In addition, while the researchers aimed to have an even gender balance in each focus group, two of the five focus groups in Glasgow included only female participants, which may have influenced the discussion. The generalisability of these findings is therefore limited, as it is difficult to assess whether these young adult participants reflect the broader population. However, this study does not claim to be representative but rather provide some insight into the influences on young adults’ food choices in two different countries.

## 5. Conclusions

The findings of this research raise a series of questions for public health, specifically in relation to improving diet in young adult populations. 

First, while the literature on the various barriers to improving food choices for young adults is important, there is less understanding about the deeper, socio-ecological narratives at play that underpin their decisions about food. What drives these narratives? How might we address them in order to change behaviour? We believe this paper offers an insight into this. 

Second, the approaches used to address poor diet tend to be individual-focused, education-based interventions. While these interventions and approaches may be well-meaning and evidence-based, they may also be promoting unhelpful attitudes about adulthood and individual choice that are disempowering and disengaging for young adults’ sense of self-efficacy, particularly for educated, urbanised young adults in high income countries. We suggest it may be more useful to explore young adults’ opinions about regulatory and policy interventions. Such research could help to identify which interventions are most acceptable to young adults. This has the potential to both increase young adults’ agency around food choices while also promoting changes to our physical and social environments to better support a sense of self-efficacy.

Third, narrative analysis is an under-utilised methodology in public health. We believe that narrative analysis, along with other less well known qualitative methods, could be used more widely to explore these questions and shed light on how different people and populations interpret and construct their experiences relating to food, diet and the prevention of non-communicable disease. 

## Figures and Tables

**Table 1:** Characteristics of focus group participants.

	Sydney	Glasgow	Total
Gender			
Female	7 (50%)	11 (69%)	18 (60%)
Male	7 (50%)	5 (31%)	12 (40%)
Age range (years)	19–29	19–29	
Average age (years)	25	24	
Age group			
18–24 years	5 (36%)	7 (44%)	12 (40%)
25–30 years	9 (64%)	9 (56%)	18 (60%)
Employment/education status			
Employed full time	5 (36%)	6 (37%)	11 (37%)
Employed part time		3 (19%)	3 (10%)
University or college student	8 (57%)	7 (44%)	15 (50%)
Other	1 (7%)	-	1 (3%)
Highest level of education completed			
High school—Year 10 or 4th form	-	-	-
High school—Year 12 or 6th form	2 (14%)	4 (25%)	6 (20%)
Bachelor degree or diploma	9 (64%)	10 (63%)	19 (63%)
Master’s degree or other postgraduate degree	3 (22%)	2 (12%)	5 (17%)
SES of home suburb ^1^			
Average	7.57	6.15	-
Median	8.50	7.00	-
Fluent (native) English speakers	11 (79%)	15 (94%)	26 (87%)
Total	14	16	30

^1^ For SES of home suburb for Glasgow participants, we cross-referenced the home suburb postcode given with the Scottish Index of Multiple Deprivation 2016 Deciles, where 1 is most deprived and 10 is least deprived [37]. For Sydney participants’ SES, we cross-referenced the home suburb given with the ABS’ State Suburb (SSC) Index of Relative Socio-economic Advantage & Disadvantage NSW, a data cube of the Socio-Economic Indexes for Areas (SEIFA). We used the decile ranking within NSW which is a SES decile score of 1 to 10, where 1 is most deprived and 10 is least deprived [38].

## Supplementary Materials

The following are available online at http://www.mdpi.com/2072-6643/10/8/996/s1, Table S1: Narrative code descriptions.

## References

[1] World Health Organization (2015). Fact Sheet No. 394: Healthy Diet.

[2] Gakidou E., Afshin A., Abajobir A.A., Abate K.H., Abbafati C., Abbas K.M., Abd-Allah F., Abdulle A.M., Abera S.F., Aboyans V. (2017). Global, regional and national comparative risk assessment of 84 behavioural, environmental and occupational and metabolic risks or clusters of risks: A systematic analysis for the Global Burden of Disease Study 2016. Lancet.

[3] Graham H. (2002). Building an inter-disciplinary science of health inequalities: The example of lifecourse research. Soc. Sci. Med..

[4] Stroud C., Walker L.R., Davis M., Irwin C.E.J. (2015). Investing in the Health and Well-Being of Young Adults. J. Adolesc. Health.

[5] Grech A., Allman-Farinelli M. (2016). Prevalence and period trends of overweight and obesity in Australian young adults. Eur. J. Clin. Nutr..

[6] Allman-Farinelli M. (2015). Nutrition Promotion to Prevent Obesity in Young Adults. Healthcare.

[7] Dietz W.H. (2017). Obesity and excessive weight gain in young adults: New targets for prevention. JAMA.

[8] Munt A.E., Partridge S.R., Allman-Farinelli M. (2017). The barriers and enablers of healthy eating among young adults: A missing piece of the obesity puzzle: A scoping review. Obes. Rev..

[9] Nikolaou C.K., Hankey C.R., Lean M.E.J. (2014). Weight changes in young adults: A mixed-methods study. Int. J. Obes..

[10] Swinburn B., Egger G., Raza F. (1999). Dissecting Obesogenic Environments: The Development and Application of a Framework for Identifying and Prioritizing Environmental Interventions for Obesity. Prev. Med..

[11] Swinburn B.A., Sacks G., Hall K.D., McPherson K., Finegood D.T., Moodie M.L., Gortmaker S.L. (2011). The global obesity pandemic: Shaped by global drivers and local environments. Lancet.

[12] Brug J., Kremers S.P., Lenthe F.V., Ball K., Crawford D. (2008). Environmental determinants of healthy eating: In need of theory and evidence: Symposium on ‘Behavioural nutrition and energy balance in the young’. Proc. Nutr. Soc..

[13] Sallis J.F., Glanz K. (2009). Physical Activity and Food Environments: Solutions to the Obesity Epidemic. Milbank Q..

[14] Furst T., Connors M., Bisogni C.A., Sobal J., Falk L.W. (1996). Food Choice: A Conceptual Model of the Process. Appetite.

[15] Sobal J., Bisogni C.A. (2009). Constructing Food Choice Decisions. Ann. Behav. Med..

[16] Plotnikoff R.C., Costigan S.A., Williams R.L., Hutchesson M.J., Kennedy S.G., Robards S.L., Allen J., Collins C.E., Callister R., Germov J. (2015). Effectiveness of interventions targeting physical activity, nutrition and healthy weight for university and college students: A systematic review and meta-analysis. Int. J. Behav. Nutr. Phys. Act..

[17] Deliens T., Clarys P., De Bourdeaudhuij I., Deforche B. (2014). Determinants of eating behaviour in university students: A qualitative study using focus group discussions. BMC Public Health.

[18] Pelletier J.E., Laska M.N. (2012). Balancing Healthy Meals and Busy Lives: Associations between Work, School and Family Responsibilities and Perceived Time Constraints among Young Adults. J. Nutr. Educ. Behav..

[19] Hebden L., Chan H.N., Louie J.C., Rangan A., Allman-Farinelli M. (2015). You are what you choose to eat: Factors influencing young adults’ food selection behaviour. J. Hum. Nutr. Diet..

[20] Freeman B., Kelly B., Vandevijvere S., Baur L. (2016). Young adults: Beloved by food and drink marketers and forgotten by public health?. Health Promot. Int..

[21] Stok F., Renner B., Clarys P., Lien N., Lakerveld J., Deliens T. (2018). Understanding Eating Behavior during the Transition from Adolescence to Young Adulthood: A Literature Review and Perspective on Future Research Directions. Nutrients.

[22] Stokols D. (1992). Establishing and Maintaining Healthy Environments: Towards a Social Ecology of Health Promotion. Am. Psychol..

[23] McLeroy K.R., Bibeau D., Steckler A., Glanz K. (1988). An ecological perspective on health promotion programs. Health Educ. Behav..

[24] Bandura A. (1986). Social Foundations of Thought and Action: A social Cognitive Theory.

[25] Nour M., Sui Z., Grech A., Rangan A., McGeechan K., Allman-Farinelli M. (2017). The fruit and vegetable intake of young Australian adults: A population perspective. Public Health Nutr..

[26] Lei L., Rangan A., Flood V.M., Louie J.C. (2016). Dietary intake and food sources of added sugar in the Australian population. Br. J. Nutr..

[27] Australian Bureau of Statistics (2012). Australian Health Survey: First Results, 2011–2012.

[28] Food Standards Scotland (2018). The Scottish Diet: It Needs to Change—2018 Update.

[29] Campbell-Jack D., Hinchliffe S., Rutherford L. (2016). The Scottish Health Survey 2015: Main Report.

[30] Liamputtong P. (2011). Focus Group Methodology: Principles and Practices.

[31] Holstein J.A., Gubrium J.F. (2014). Handbook of Constructionist Research.

[32] Hurwitz B., Greenhalgh T., Skultans V. (2004). Narrative Research in Health and Illness.

[33] Greenhalgh T., Wengraf T. (2008). Collecting stories: Is it research? Is it good research? Preliminary guidance based on a Delphi study. Med. Educ..

[34] Liamputtong P. (2010). Research Methods in Health: Foundation for Evidence-Based Practice.

[35] Thornberg R., Charmaz K., Flick U. (2013). Grounded Theory and Theoretical Coding. The SAGE Handbook of Qualitative Data Analysis..

[36] Braun V., Clarke V. (2013). Successful Qualitative Research: A Practical Guide for Beginners.

[37] Scottish Government (2016). The Scottish Index of Multiple Deprivation (SIMD) 2016.

[38] Australian Bureau of Statistics (2018). Socio-Economic Indexes for Areas (SEIFA) 2016. http://www.abs.gov.au/ausstats/abs@.nsf/mf/2033.0.55.001.

[39] Davison J., Share M., Hennessy M., Bunting B., Markovina J., Stewart-Knox B. (2015). Correlates of food choice in unemployed young people: The role of demographic factors, self-efficacy, food involvement, food poverty and physical activity. Food Q. Prefer..

[40] Neely E., Walton M., Stephens C. (2014). Young people’s food practices and social relationships. A thematic synthesis. Appetite.

[41] Venn D., Dixon J., Banwell C., Strazdins L. (2018). Social determinants of household food expenditure in Australia: The role of education, income, geography and time. Public Health Nutr..

[42] Venn D., Strazdins L. (2017). Your money or your time? How both types of scarcity matter to physical activity and healthy eating. Soc. Sci. Med..

[43] Thornton L.E., Cameron A.J., McNaughton S.A., Worsley A., Crawford D.A. (2012). The availability of snack food displays that may trigger impulse purchases in Melbourne supermarkets. BMC Public Health.

[44] Mhurchu C.N., Vandevijvere S., Waterlander W., Thornton L.E., Kelly B., Cameron A.J., Snowdon W., Swinburn B. (2013). Monitoring the availability of healthy and unhealthy foods and non-alcoholic beverages in community and consumer retail food environments globally. Obes. Rev..

[45] Freeman B., Kelly B., Baur L., Chapman K., Chapman S., Gill T., King L. (2014). Digital Junk: Food and Beverage Marketing on Facebook. Am. J. Public Health.

[46] McNaughton M.J. (2008). Guerrilla communication, visual consumption and consumer public relations. Public Relat. Rev..

[47] Carter S.M. (2014). Health promotion: An ethical analysis. Health Promot. J. Austr..

[48] Carter S.M., Rychetnik L., Lloyd B., Kerridge I.H., Baur L., Bauman A., Hooker C., Zask A. (2011). Evidence, Ethics and Values: A Framework for Health Promotion. Am. J. Public Health.

[49] Pelletier J.E., Laska M.N., Neumark-Sztainer D., Story M. (2013). Positive Attitudes toward Organic, Local and Sustainable Foods Are Associated with Higher Dietary Quality among Young Adults. J. Acad. Nutr. Diet..

[50] Howse E., Freeman B., Wu J.H.Y., Rooney K. (2017). ‘The university should promote health but not enforce it’: Opinions and attitudes about the regulation of sugar-sweetened beverages in a university setting. BMC Public Health.

[51] Grunseit A.C., Rowbotham S., Crane M., Indig D., Bauman A., Wilson A. (2018). Nanny or canny? Community perceptions of government intervention for preventive health. Crit. Public Health.

[52] Hawkes C., Smith T.G., Jewell J., Wardle J., Hammond R.A., Friel S., Thow A.M. (2015). Smart food policies for obesity prevention. Lancet.

[53] Doherty S., Cawood J., Dooris M. (2011). Applying the whole-system settings approach to food within universities. Perspect. Public Health.

