# Supplementary material for: ‘Buying Salad Is a Lot More Expensive than Going to McDonalds’: Young Adults’ Views about What Influences Their Food Choices
Source: Nutrients. 2018 Jul 30;10(8):996. doi: 10.3390/nu10080996 (PMC6116192; doi:10.3390/nu10080996)
Supplement: Supplementary file 1 [file nutrients-10-00996-s001.pdf]

Supplementary File

Table S1. Narrative code descriptions.

**Supplementary File, Table S1: Narrative code descriptions. Narrative code**	**Description of narrative code**	**Link to main narrative**
**Price and cost**	Food choices are influenced by pricing of food ie. Prioritising foods that cost less. Also food choices that represent value for money (ie. Filling / address hunger given the cost).	Value of food
**Placement**	Food choices are influenced by the placement and presentation of foods, such as in supermarkets and stores.	Appeal of food
**Flavour**	Food choices are influenced by an enjoyment or recognition of taste.	Appeal of food
**Time**	Food choices are influenced by time or lack thereof – foods are prioritised if they are quick and convenient (cost less in terms of time).	Value of food
**Availability**	Food choices are influenced by what is most accessible and available to buy.	Appeal of food
**Novelty**	Food choices are influenced by the novelty or newness of products.	Appeal of food
**Emotions**	Food choices are influenced by a specific emotional connection, such as: wanting to 'feel good' or cheer up (comfort); memory/nostalgia of childhood and other places; family / community / love; guilt and shame.	Emotional connections with food
**Culture**	Food choices are influenced by a specific cultural group or cultural practices eg. Valuing ethnic groups and their specific cuisines.	Emotional connections with food
**Life stage**	Food choices are influenced by a specific life stage or period of life eg. Being a kid; going to university; moving out of home; working full time OR not working at all.	Value of food
**Purpose**	Food choices are influenced by association of that food or drink with a particular purpose or action.	Appeal of food
**Habit**	Food choices are influenced by well-established patterns or habits.	Appeal of food
**Reliability**	Food choices are influenced by the knowledge of their reliability - choosing certain foods because you know what they taste like.	Appeal of food
**Versatility**	Food choices are influenced by whether or not they are versatile and not boring.	Value of food
**Brands**	Mentioning of specific food, supermarket or other brands.	Appeal of food
**Ethical and moral considerations**	Food choices are influenced by ethical and moral considerations including: animal rights/vegetarianism; sustainability and climate change; mass food production etc.	Emotional connections with food

**Food trends**	Food choices are influenced by broader food trends such as gluten free; low sugar/low carb; organic foods.	Other
**Specific health needs**	Food choices are influenced by specific health reasons or diets, including ‘medical’ diets.	Other
**Advertising & marketing**	Food choices are influenced by advertising, marketing or promotions, including by celebrities on social media.	Appeal of food
**Social norms**	Food choices are influenced by social and behavioural norms including the influence of peers.	Emotional connections with food
**Performing adulthood**	Food choices are influenced by the notion of being a ‘good’ adult and making ‘good’ choices around food. Includes critical reflection of decision-making and choices.	Performing adulthood
